# Upregulation of C1-inhibitor in pancreatic cancer

**DOI:** 10.18632/oncotarget.27191

**Published:** 2019-10-01

**Authors:** Kurt Osther, Karolina Förnvik, Emma Liljedahl, Leif G. Salford, Henrietta Nittby Redebrandt

**Affiliations:** ^1^The Rausing Laboratory, Division of Neurosurgery, Department of Clinical Sciences, Lund University, Lund, Sweden; ^2^Department of Clinical Chemistry, Skåne University Hospital, Scania, Sweden; ^3^Department of Neurosurgery, Skåne University Hospital, Scania, Sweden

**Keywords:** complement system, C1-inhibitor, pancreatic cancer, immunotherapy, cancer

## Abstract

**Purpose:**

The complement system has recently sparked more interest in cancer research. The classical pathway is initiated by activation of the C1 complex, which irreversibly can be bound to and inhibited by C1-INH. We have previously shown that C1-INH is upregulated in human glioblastoma (astrocytoma grade IV) on both gene and protein level. We here examine whether the complement system seems to play a role also in pancreatic cancer.

**Technique and results:**

We performed an expression analysis of complement associated genes in 36 pancreatic ductal adenocarcinoma tumors and matching normal pancreatic tissue samples from pancreatic cancer patients (data from the publicly available database GSE15471). C1-INH was significantly upregulated in the pancreatic cancer tissue. None of the downstream components of the cascade were significantly upregulated in the cancer samples as compared to the control samples, which is the same pattern as we found in glioblastoma. GO analysis showed that membrane attack complex came up as the second most significantly associated cellular component. Analyzing gene expression of C1-INH in the pancreatic cancer cell lines from primary tumors versus metastatic tumor revealed no difference for the two mRNA transcripts (GSE59357).

**Interpretation:**

Analysis of gene expression of complement related genes shows an upregulation of C1-INH and a downregulation of downstream components. This could suggest that C1-INH plays a role also in pancreatic cancer.

## INTRODUCTION

The complement system has recently sparked more interest in cancer research. The complement system comprises three biochemical pathways; the classical, the alternative, and the lectin induced pathways. Under normal conditions the complement system acts as a functional bridge between the innate and the adaptive immune responses [1]. The classical pathway is initiated by activation of the C1 complex, which irreversibly can be bound to and inhibited by C1-INH (C1-Inhibitor, also denoted C1-inactivator (C1-IA) or Serping 1), the only known physiological inhibitor of the C1r and C1s proteases [2]. Besides its inhibitor activity in the complement system, C1-INH is also known to inhibit proteases of the fibrinolytic, clotting, and kinin pathways and additionally it is the most important physiological inhibitor of plasma kallikrein, fXIa, and fXIIa.

There is growing evidence of the presence of complement components and several regulatory proteins in most types of tumor cells [3]. We have previously shown that C1-INH is upregulated in human glioblastoma (astrocytoma grade IV) on both gene and protein level [4]. We could also demonstrate a significantly increased survival *in vivo* in rats inoculated intracerebrally with glioma cells pre-coated with anti-C1-INH antibodies [4]. Furthermore, intratumoral treatment in a subcutaneous glioblastoma model lead to smaller tumors and increased survival [5].

We here examine whether the complement system seems to play a role in pancreatic cancer, which, together with glioblastoma, is one of the most lethal forms of cancer. Pancreatic cancer has a dismal prognosis with only 5% five-year overall survival rate, which is partially attributed to the fact that the cancer is usually diagnosed at a late stage [6]. As in the glioblastoma situation, new targets are needed.

We analyze whether complement related genes are differently regulated in pancreatic cancer as compared to adjacent non-tumor tissue. Furthermore, complement related genes are compared in primary versus metastatic tumor cell lines, to see if there is any indication that the complement system is involved in metastatic properties of this tumor. Drug related expression of C1-INH is also analyzed with regards to possible new targets that could be used against pancreatic cancer. Chemotherapeutic treatment of pancreatic cancer not cured by surgery is today a major problem. Screening of possible therapeutic targets, as done by Chien et al. [7], might reveal promising drugs. Chien et al. [7] analyzed 66 kinase inhibitors on pancreatic cancer cell lines and found that most of the cell lines were resistant to most of the kinase inhibitors, as compared to hematologic malignancies for instance. However, some kinase inhibitors seemed to be useful. Dasatinib, an RTK/SRC/TEC inhibitor, was the most potent small molecule inhibitor on the pancreatic cancer cells and divided the cells into sensitive ones and resistant ones. We analyzed C1-INH in relation to Dasatinib sensitivity to see whether expression of this gene was related to drug sensitivity. Chien et al. [7] also presented that PP2A, protein phosphatase 2 catalytic subunit alpha, a tumor suppressor protein, might be a possible target in pancreatic cancer, and reported that activating this gene together with administration of the drug Penfluridol increased sensitivity of pancreatic cancer cells to this treatment. PP2A accounts for serine–threonine phosphatase activity in eukaryotic cells and studies have shown that inhibition of PP2A expression and/or function may contribute to leukemogenesis in several hematological malignancies [8]. We here analyze the expression of PP2A in pancreatic cancer tissue versus control tissue and compare the fold change to that seen in our suggested target protein C1-INH. Finally, we seek in the public database Protein Atlas for clinical correlation of C1-INH expression and survival in patients with pancreatic cancer [9].

## RESULTS

### Gene expression of complement related components revealed increased C1-INH in tumor versus control tissue

**Figure 1:**
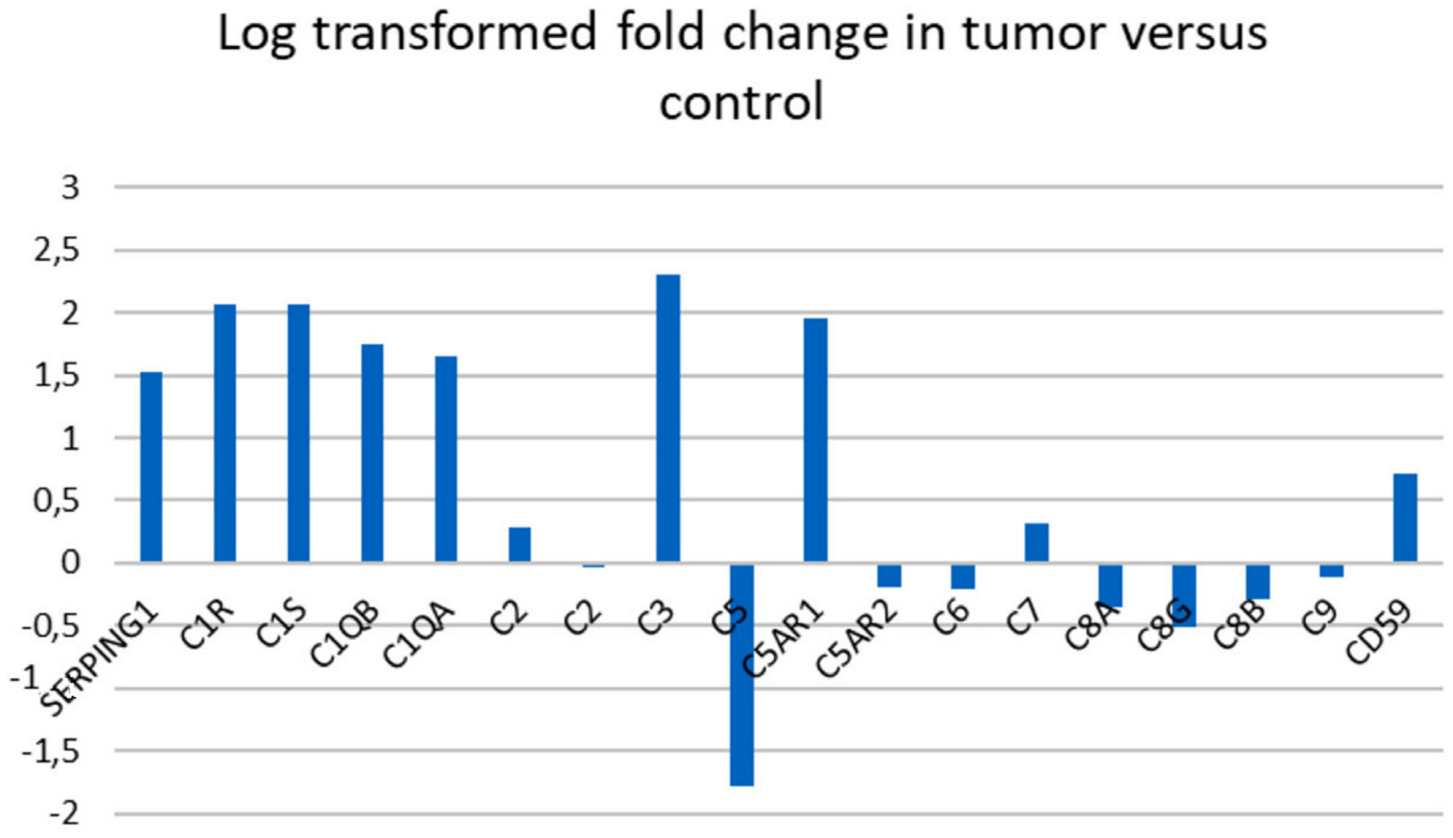
Gene expression in complement associated genes in pancreatic cancer versus control tissue. Log transformed fold change in 18 complement associated genes comparing tumor tissue to control tissue. A log transformed fold change >1 was considered significant. Complement component C2 was represented with two different annotations.

We performed an expression analysis of complement associated genes in the 36 pancreatic ductal adenocarcinoma tumors and matching normal pancreatic tissue samples from pancreatic cancer patients (GSE15471) [10]. 18 genes associated with the complement cascade were analyzed. Table 1 and Figure 1 present the list of the genes and log transformed fold changes found comparing pancreatic cancer tissue to control tissue. C1-INH was significantly upregulated in the pancreatic cancer tissue. None of the downstream components of the cascade were significantly upregulated in the cancer samples as compared to the control samples, which is the same pattern as we found in glioblastoma [4].

**Table 1:** Analyses of differences in expressions in genes correlated to the complement cascade

IDENTIFIER	Log Fold change	*t*-test
**SERPING1**	**1,521764359**	**6,52541E-13**
C1R	2,060787692	4,43095E-12
C1S	2,064776923	3,27155E-09
C1QB	1,750484359	7,26041E-06
C1QA	1,654581538	2,40136E-05
C2	0,276473846	0,034250962
C2	-0,029108974	0,617523946
C3	2,309411026	3,4795E-11
C5	-1,788636154	2,65049E-08
C5AR1	1,962011538	1,77589E-08
C5AR2	-0,186818974	0,001275694
C6	-0,211024359	0,609480915
C7	0,318995128	0,212537837
C8A	-0,346529744	2,27017E-07
C8G	-0,51640359	6,02514E-05
C8B	-0,283583846	0,000155076
C9	-0,116497436	0,00077029
CD59	0,715847949	4,90319E-08

C1-INH (Serping 1) was significantly up-regulated in the pancreatic cancer tissues as compared to the control tissues. Six other genes were also significantly upregulated in the tissues with pancreatic cancer, with log fold change > 1. None of the downstream components of the cascade were upregulated (C6, C7, C8 or C9).

### GO analysis where membrane attack complex was an associated cellular component

**Figure 2:**
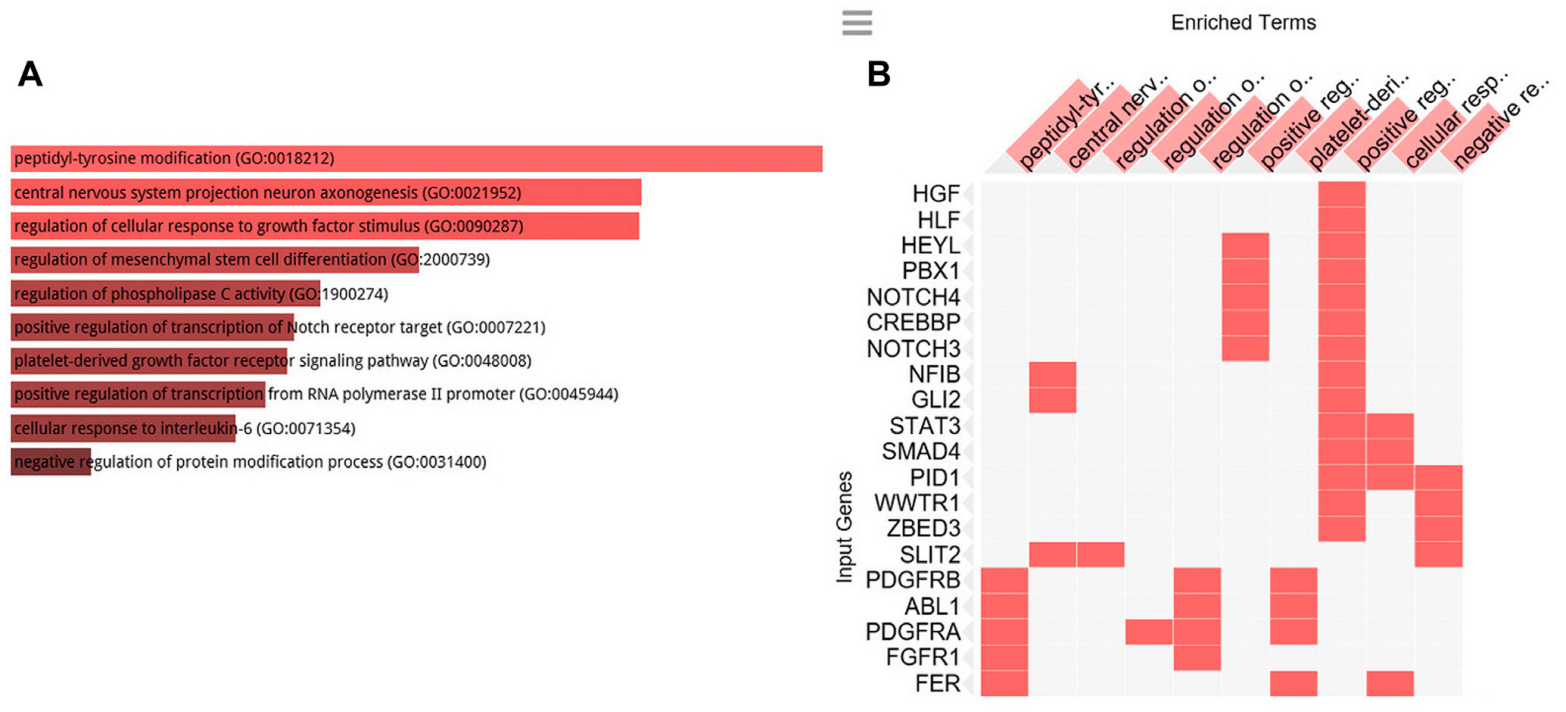
GO Biological processes analysis. GO biological processes associated with the genes positively correlated to C1-INH. (**A**) Bar graph of pathways associated with C1-INH, sorted by *p*-value ranking. (**B**) Clustergram of pathways associated with C1-INH. Enriched terms are the columns, input genes are the rows, and cells in the matrix indicate if a gene is associated with a term. From Enrichr (http://amp.pharm.mssm.edu/Enrichr/).

**Figure 3:**
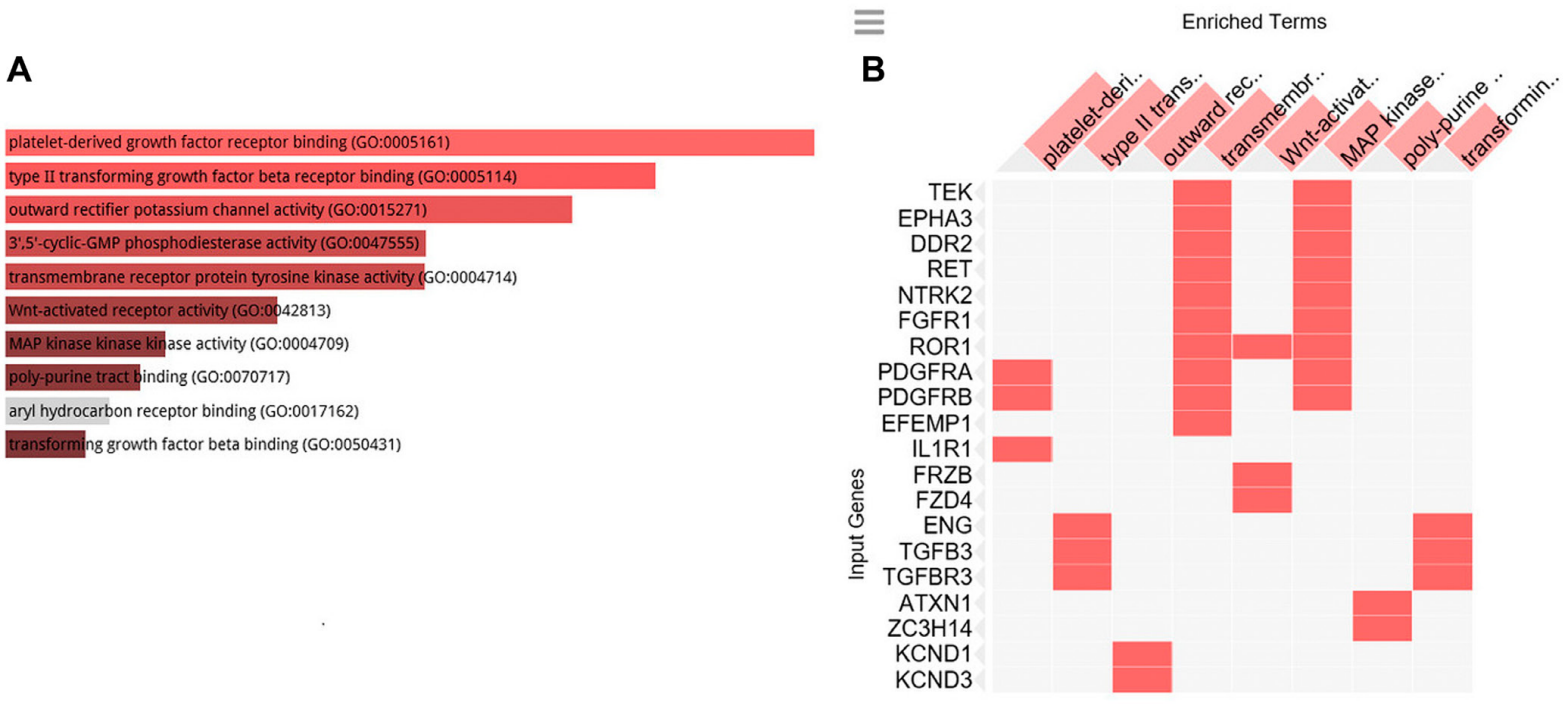
GO cellular function analysis. GO cellular function associated with the genes positively correlated to C1-INH. (**A**) Bar graph of pathways associated with C1-INH, sorted by *p*-value ranking. (**B**) Clustergram of pathways associated with C1-INH. Enriched terms are the columns, input genes are the rows, and cells in the matrix indicate if a gene is associated with a term. From Enrichr (http://amp.pharm.mssm.edu/Enrichr/).

**Figure 4:**
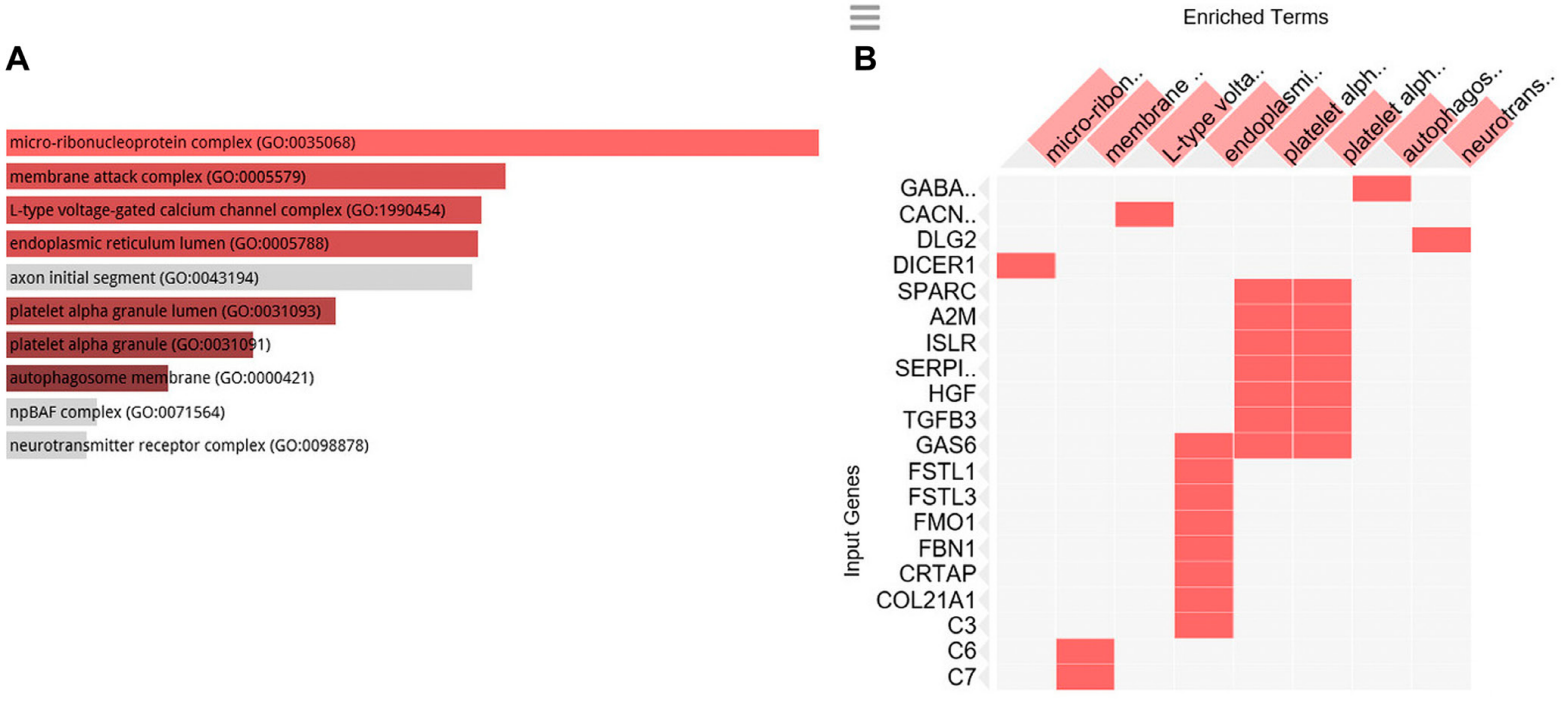
GO cellular component analysis. GO cellular component associated with the genes positively correlated to C1-INH. (**A**) Bar graph of pathways associated with C1-INH, sorted by *p*-value ranking. (**B**) Clustergram of pathways associated with C1-INH. Enriched terms are the columns, input genes are the rows, and cells in the matrix indicate if a gene is associated with a term. From Enrichr (http://amp.pharm.mssm.edu/Enrichr/).

To clarify the biological role of C1-INH in pancreatic cancer, we performed GO analysis. Firstly, we created a gene list that strongly correlated with C1-INH (SERPING1) by Pearson correlation analysis (Pearson > 0.5). Then, we explored the biological process of these genes by GO analysis through Enrichr (http://amp.pharm.mssm.edu/Enrichr/). The top 10 GO terms associated with biological processes are shown in Figure 2A and a cluster map is presented in Figure 2B, *p*-values are presented in Table 2. The top 10 GO terms associated with molecular functions are shown in Figure 3A and a cluster map is presented in Figure 3B, *p*-values are presented in Table 3. The top 10 GO terms associated with cellular components are shown in Figure 4A and a cluster map is presented in Figure 4B, *p*-value are presented in Table 4. Here membrane attack complex came up as the second most significantly associated cellular component.

**Table 2:** GO Biological process 2018 associated with genes positively correlated to C1-INH, showing top 10 of 2,630 entries, *p*-value calculated from Fisher exact probability test

Index	Name	*P*-value
1	peptidyl-tyrosine modification (GO:0018212)	0.000008659
2	central nervous system projection neuron axonogenesis (GO:0021952)	0.0008154
3	regulation of cellular response to growth factor stimulus (GO:0090287)	0.0009330
4	regulation of mesenchymal stem cell differentiation (GO:2000739)	0.002299
5	regulation of phospholipase C activity (GO:1900274)	0.0008154
6	positive regulation of transcription of Notch receptor target (GO:0007221)	0.0007120
7	platelet-derived growth factor receptor signaling pathway (GO:0048008)	0.0007120
8	positive regulation of transcription from RNA polymerase II promoter (GO:0045944)	0.0002074
9	cellular response to interleukin-6 (GO:0071354)	0.0003860
10	negative regulation of protein modification process (GO:0031400)	0.0003222

**Table 3:** GO Cellular functions 2018 associated with genes positively correlated to C1-INH, showing top 10 of 541 entries, *p*-value calculated from Fisher exact probability test

Index	Name	*P*-value
1	platelet-derived growth factor receptor binding (GO:0005161)	0.002235
2	type II transforming growth factor beta receptor binding (GO:0005114)	0.003564
3	outward rectifier potassium channel activity (GO:0015271)	0.002947
4	3′,5′-cyclic-GMP phosphodiesterase activity (GO:0047555)	0.01234
5	transmembrane receptor protein tyrosine kinase activity (GO:0004714)	0.0002467
6	Wnt-activated receptor activity (GO:0042813)	0.002347
7	MAP kinase kinase kinase activity (GO:0004709)	0.005917
8	poly-purine tract binding (GO:0070717)	0.01057
9	aryl hydrocarbon receptor binding (GO:0017162)	0.05247
10	transforming growth factor beta binding (GO:0050431)	0.008849

**Table 4:** GO Cellular component 2018 associated with genes positively correlated to C1-INH, showing top 10 of 219 entries, *p*-value calculated from Fisher exact probability test

Index	Name	*P*-value
1	micro-ribonucleoprotein complex (GO:0035068)	0.04196
2	membrane attack complex (GO:0005579)	0.03236
3	L-type voltage-gated calcium channel complex (GO:1990454)	0.04196
4	endoplasmic reticulum lumen (GO:0005788)	0.005544
5	axon initial segment (GO:0043194)	0.06379
6	platelet alpha granule lumen (GO:0031093)	0.02409
7	platelet alpha granule (GO:0031091)	0.01447
8	autophagosome membrane (GO:0000421)	0.04370
9	npBAF complex (GO:0071564)	0.08855
10	neurotransmitter receptor complex (GO:0098878)	0.1018

### No significant increase of C1-INH in metastatic versus primary pancreatic tumor cells

Analyzing gene expression of C1-INH in the pancreatic cancer cell lines from primary tumors versus metastatic tumor revealed no difference for the two mRNA transcripts analyzed in the platform (*t*-test n. s.). C5 was the only complement related component which was significantly different in the primary tumors versus metastatic cells (*p*-value = 0.02), however with a fold change of only 0.5.

### Increased expression of C1-INH in Dasatinib sensitive versus resistant tumors

**Figure 5:**
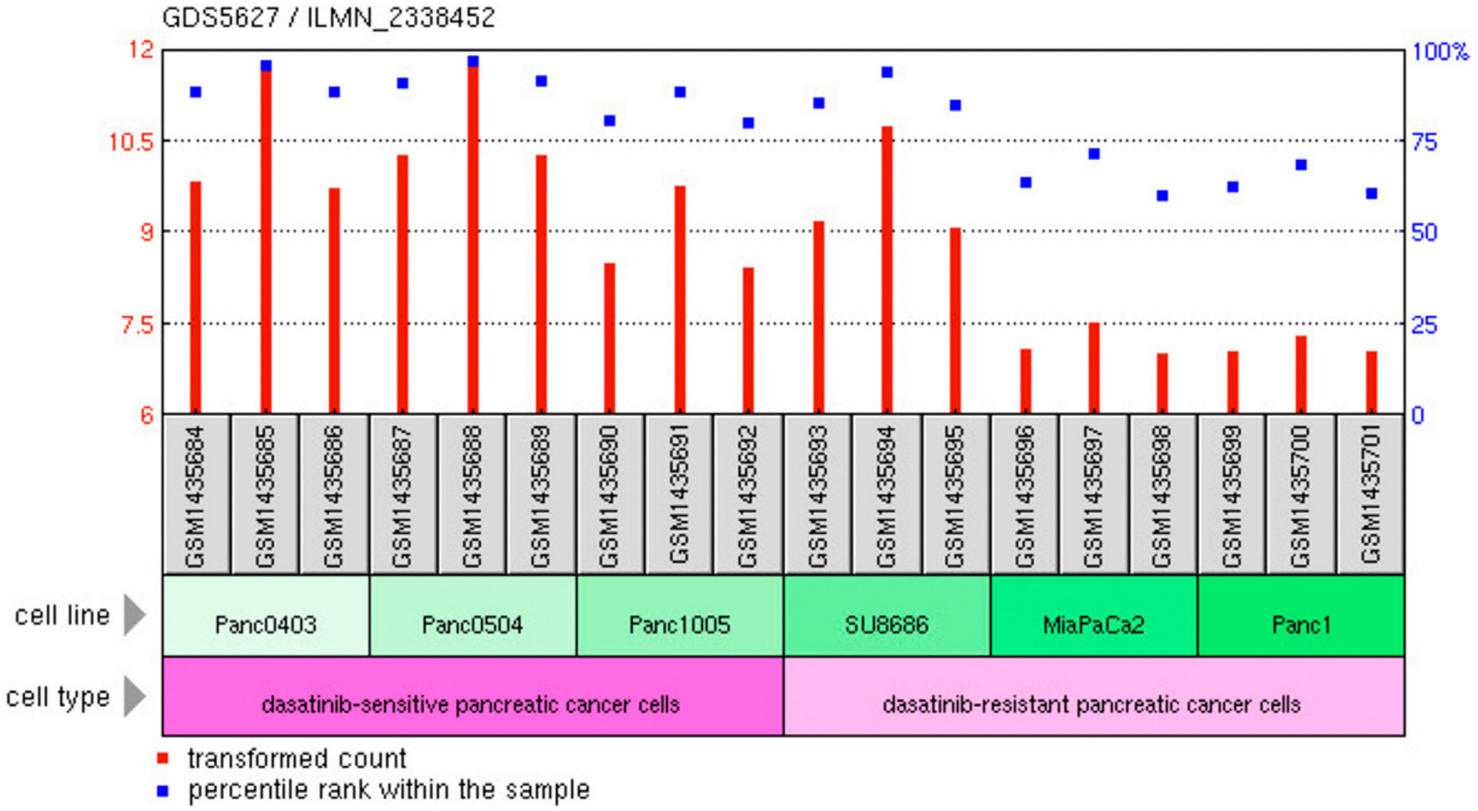
C1-INH versus Dasatinib sensitivity. Expression of C1-INH in Dasatinib sensitive versus resistant pancreatic cancer cells. From https://www.ncbi.nlm.nih.gov/geo/tools/.

Analyzing gene expression of C1-INH in the pancreatic cancer cell lines from Dasatinib sensitive versus resistant pancreatic tumor cells lines using mRNA transcripts showed that C1-INH was significantly upregulated in Dasatinib sensitive versus resistant tumor cell lines (*p*-value 0.004, fold change 2), with a higher expression of C1-INH in the sensitive cell lines as compared to the resistant ones (Figure 5).

### C1-INH is significantly altered between tumor and control tissue, and significantly higher expressed in tumors as compared to PP2A

**Figure 6:**
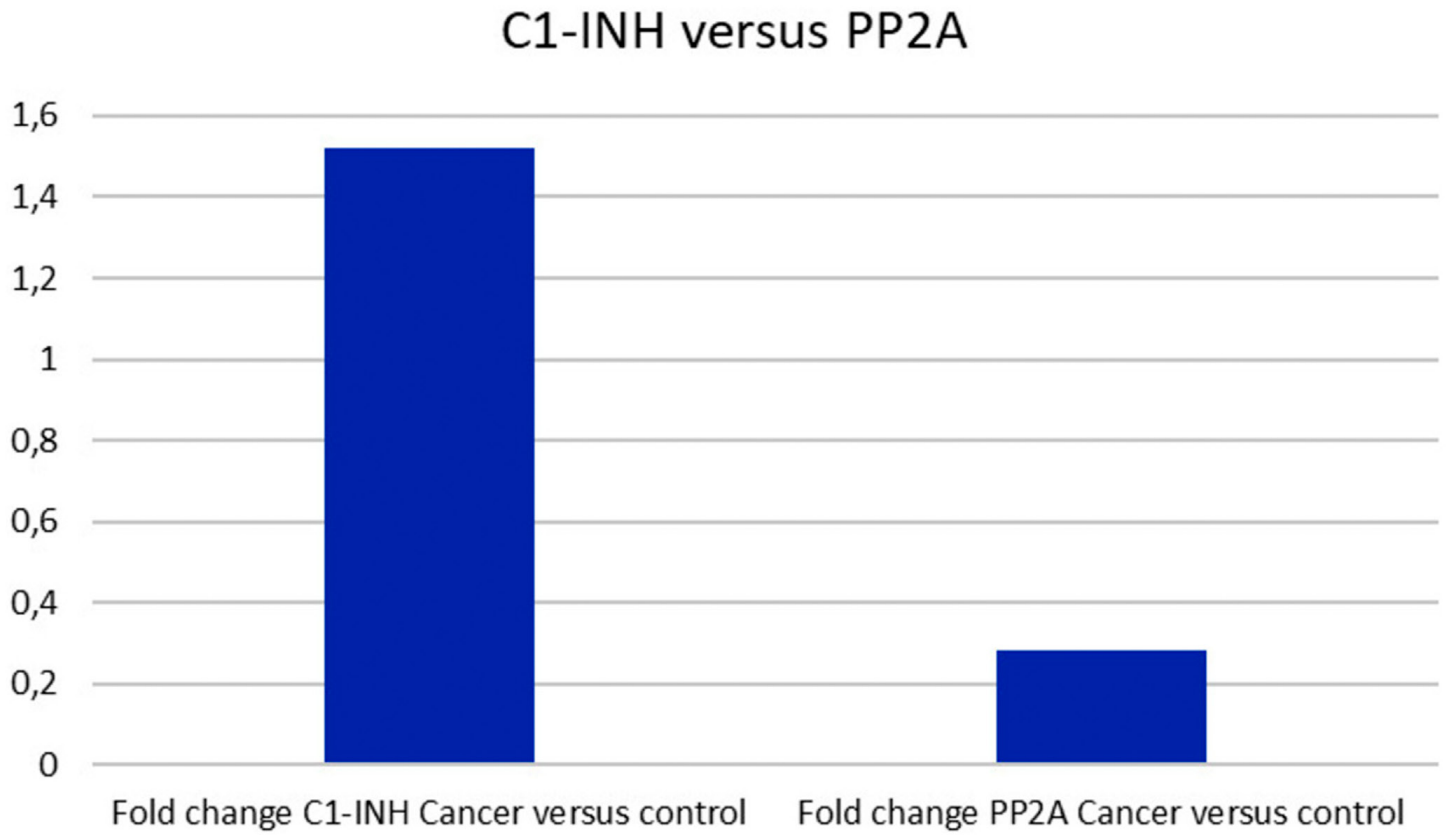
C1-INH versus PP2A expression. Whereas C1-INH was differently expressed in pancreatic cancer as compared to control tissue, no difference was seen regarding PP2A.

Comparing the expression of C1-INH to that of PP2A, with regards to the fold change in tumor versus control, C1-INH was significantly increased in tumors, as reported above. PP2A on the other hand, had no significant increase in fold change in tumors as compared to control tissue (Figure 6). This might indicate that targeting PP2A could be problematic, since side effects are more likely to be expected as suggested by the results generated here.

### High expression of C1-INH is correlated to poor survival in patients with pancreatic cancer

**Figure 7:**
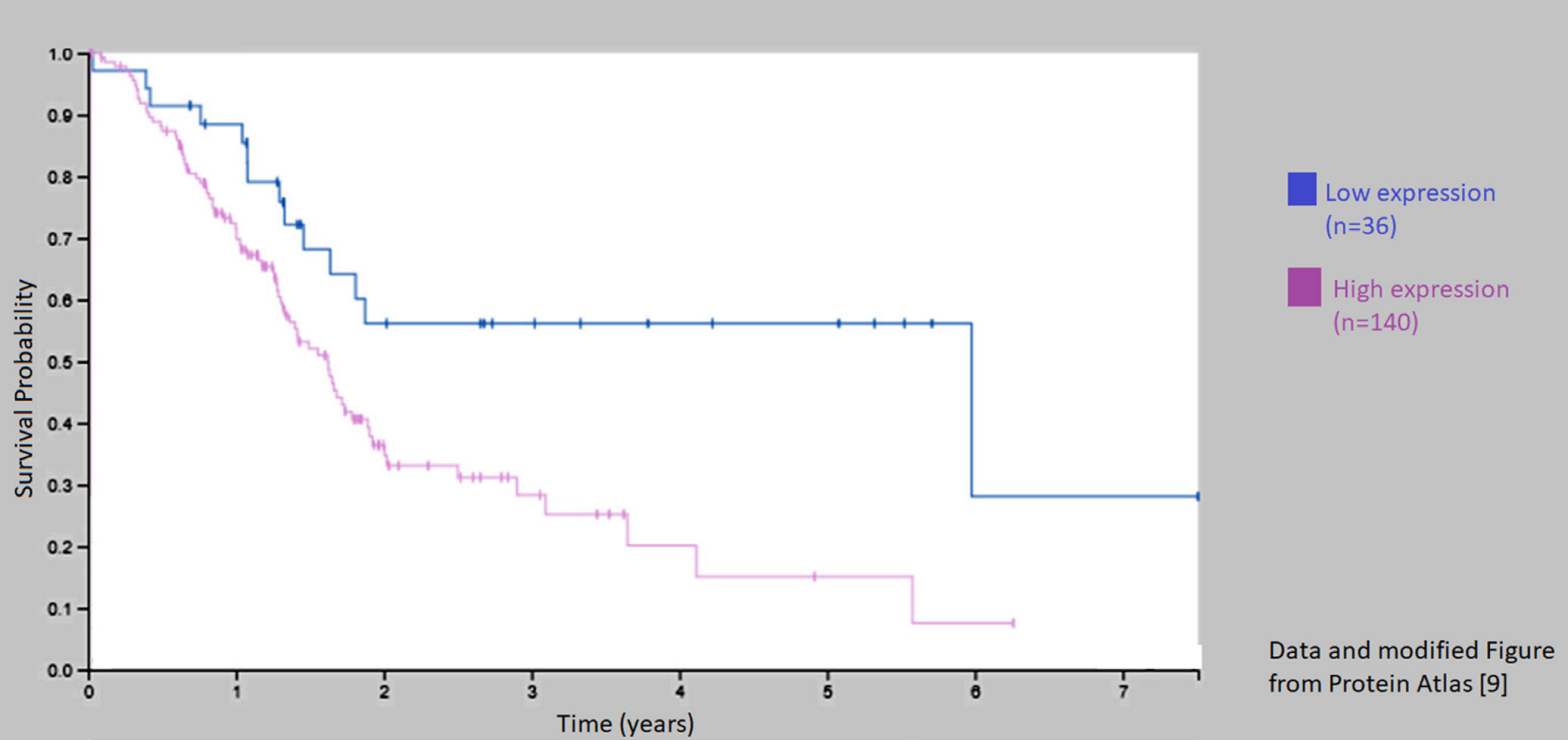
Kaplan–Meier curve for patients with low and high expression of C1-INH (Sering 1). Survival was decreased in those patients with a high expression of C1-INH (Serping 1) as compared to those with a low expression from Protein Atlas [9].

Data from proteinatlas. org, Pathology atlas [9] (https://www.proteinatlas.org/ENSG00000149131-SERPING1/pathology) showed that 5-year survival was 15% in patients with pancreatic cancer and high expression of Serping 1 (C1-INH), whereas those with a low expression had a 5-year survival of 56% (p-score 0.0059) (Figure 7). With the antibody HPA048738 91.6% of the analyzed pancreatic tumors showed a high/medium expression of C1-INH. Also several cases of endometrial, thyroid, lung, testicular and liver cancers exhibited strong immunoreactive for Serping 1 with this antibody, whereas glioma and lymphomas had 25% and 30% respectively of high/medium expression. Using the other antibody CAB026161, only few patients had positive staining in the cancer forms analyzed, suggesting that this clonality of the monoclonal antibody might not be optimal.

## DISCUSSION

We have recently described that C1-INH, is overexpressed in glioblastoma tissue on gene level and protein level, and on glioblastoma cells from patients and rat glioma cell lines [4], which introduces C1-INH as a potentially important factor in glioblastoma research. Following the analyses described above, it seems like a similar pattern could be suggested on gene level also in pancreatic carcinoma. We found a similar upregulation if C1-INH in tumor tissue as compared to control tissue. Furthermore, downregulation of down-stream components of the complement cascade was seen in pancreatic cancer in the same way as in glioblastoma. Data from Protein Atlas [9] suggest that a high expression of C1-INH is correlated to poor 5-year survival; and furthermore, that C1-INH can be detected in a large proportion of samples collected from patients with pancreatic cancer.

We also analyzed C1-INH in relation to suggested novel targets against pancreatic cancer. We could see that C1-INH expression correlated to Dasatinib sensitive pancreatic cancer cells. We could also see that it was significantly differently expressed in tumor tissue as compared to control tissue, which was in clear contrast to PP2A, which has been suggested to be a target to be activated in conjunction to therapies [7]. Furthermore, there was no significant difference in the expression of complement associated genes, including C1-INH, in tumor versus metastatic pancreatic cancer cells. This seems to be useful, since pancreatic cancer often is metastasized already when the disease is diagnosed.

Despite the development of immunotherapies as promising treatment choices in other forms of cancer, there has been limited success in pancreatic cancer, as well as glioblastoma. Pancreatic cancer has been unresponsive to both anti-programmed death 1 (anti-PD-1) and anti-cytotoxic T-lymphocyte-associated antigen 4 (anti-CTLA-4) [11, 12]. The explanation to these unsatisfying results may lay in the quite unique tumor microenvironment seen in pancreatic cancer. An excessive stromal matrix and hypo-vascularity creates a tumor microenvironment containing strong inhibitory signaling circuits and massive physical barriers for immune agent infiltration [13].

The role of the complement system in pancreatic cancer needs to be explored further. There are only few reports focusing on this at the present stage. Shi et al. [14] reported that complement component 1, q subcomponent binding protein (C1QBP), in lipid rafts mediates hepatic metastasis of pancreatic cancer by regulating IGF-1/IGF-1R signaling. They reported that many human cancers exhibit higher C1QBP expression levels than their nonmalignant histologic counterparts, including thyroid, lung, esophagus, gastric and colon cancer and referred to that previous reports have confirmed that C1QBP mediates epidermal growth factor (EGF)-induced cancer cell chemotaxis and distant metastasis by activation of receptor tyrosine kinases.

It would be interesting to analyze further whether components of the complement system could serve as prognostic markers. In pancreatic cancer, tumor marker CA 19-9 seems to have a value as a prognostic factor, as it may be used to measure disease burden and potentially guide treatment decisions. For example, a serum level ≥500 UI/ml CA 19-9 preoperatively, indicated a poorer prognosis after surgery [15]. C3 and soluble iC3b have also been suggested as tumor markers for pancreatic cancer [16, 17]. Märten et al. [16] showed that soluble iC3b plasma levels were increased up to four months before radiological evidence of disease. Chen et al. [17] described that the expression levels of complement C3, complement C4b1 and apoE were higher in pancreatic cancer cells compared to normal pancreatic tissues. However, no correlations were observed between complement C3 and tumor TNM staging, instead complement C4b1 and apoE were correlated with tumor TNM staging.

In future studies, the role of C1-INH can be explored further using cell experiments and animal trials. Possible ways to target C1-INH could be to administer polyclonal or monoclonal antibodies intravenously. Nanobodies could be tested, with the possibility to use bivalent nanobodies if needed. Regarding the correlation between C1-INH and Dasatinib sensitivity, C1-INH could theoretically be used as a marker to select tumor cells more sensitive to this kind of treatment.

## MATERIALS AND METHODS

### Analyses of complement related genes

The first set of data was retrieved from a publicly available database (GSE15471 Affymetrix Gene Chip Analysis) [10]. Combined gene expression analysis of whole-tissue and microdissected pancreatic ductal adenocarcinoma identifies genes specifically overexpressed in tumor epithelia [10]. This set of data was performed using the Affymetrix U133 plus 2.0 whole genome microarrays (54675 probesets), where pairs of normal and tumor tissue samples had been obtained at the time of surgery from resected pancreas of 36 pancreatic cancer patients. The data had already been normalized using the Robust Microarray Analysis (RMA) algorithm. The log-transformed data as produced by the RMA algorithm was used for all subsequent statistical tests. An unpaired *t*-test was used to determine the probe sets (genes) that are differentially expressed between the normal and the tumor tissue samples assuming unequal variances. A log-transformed fold-change > 1 (roughly corresponding to a fold change > 2 as described by Badea et al. [10]) and *p*-value < 0.05 were considered statistically significant.

### Gene Ontology (GO) analysis

After Pearson correlation analysis, Gene Ontology analysis of the positively correlated genes (r > 0.5) were analyzed by Enrichr (http://amp.pharm.mssm.edu/Enrichr/).

### Expression in metastasis versus primary tumor

The second set of data was retrieved from a publicly available database (Reference Series: GSE59357, Chien W, Sun QY, Lee KL, Ding LW et al. Activation of protein phosphatase 2A tumor suppressor as potential treatment of pancreatic cancer. Mol Oncol 2015 Apr; 9(4):889-905. PMID: 25637283) [7]. This set of data was performed using the GPL10558: Illumina HumanHT-12 V4.0 expression beadchip platform, analyzing mRNA expression in Dasatinib-resistant and Dasatinib-sensitive pancreatic cancer cell lines from primary tumor (Panc0403, Panc0504, Panc1005, MiaPacCa2, Panc1) and liver metastasis of pancreatic cancer (SU8686). An unpaired *t*-test assuming unequal variances was used to determine whether complement related genes were differentially expressed between the primary tumor versus metastatic tumor.

### C1-INH in dasitinib sensitive versus resistant tumors

Data was retrieved from the database with reference series GSE59357 as described above. Expression of C1-INH (annotation Serping 1 in the data set) was compared between the Dasatinib sensitive and resistant cell lines using un-paired *t*-test assuming unequal variances.

### C1-INH in relation to PP2A expression

The first set of data (GSE15471) was analyzed comparing expression between C1-INH and PP2A, using the data normalized by the Robust Microarray Analysis (RMA) algorithm. A log-transformed fold-change > 1 (roughly corresponding to a fold change < 2 as described by Badea et al. [10]) was considered significant.

### C1-INH expression in tumor samples analyzed from protein atlas

Data from proteinatlas. org was analyzed with regards to expression of C1-INH (Serping 1) [9]. Here, protein expression of C1-INH in different cancer tissues was presented, where they had used two different antibodies, HPA048738 (polyclonal, Sigma-Aldrich) and CAB026161 (monoclonal, R&D Systems).

## CONCLUSIONS

We here present data showing that C1-INH is upregulated on gene level in pancreatic cancer, together with upstream components of the complement cascade. Downstream components, including C6, C7, C8 and C9 however, were not upregulated. Analyses from databases showed that an upregulation of C1-INH was correlated to poor survival in patients with pancreatic cancer. Future *in vivo* studies targeting C1-INH in animals with pancreatic cancer would be of value.

## Abbreviations

C1-INH: C1-inhibitor
